# Supplementary material for: A set of multi-entry identification keys to African frugivorous flies (Diptera, Tephritidae)
Source: Zookeys. 2014 Jul 24;(428):97–108. doi: 10.3897/zookeys.428.7366 (PMC4143993; doi:10.3897/zookeys.428.7366)
Supplement: Supplementary material 6 — Key to Ceratitis [file zookeys-428-097-s006.zip › SF6_ZooKeys_key to Ceratitis/key/SF6_ZooKeys_key to Ceratitis/Media/Html/Ceratitis millicentae.htm]

***Ceratitis*** (***Pardalaspis***) ***millicentae***
De Meyer & Copeland

*Ceratitis* (*Pardalaspis*)
*millicentae* De Meyer & Copeland, 2005 : 1295.

Body
length. 6.54 (5.75-7.20) mm; wing length. 5.99 (5.25-6.50) mm.

Male

Head.
Antenna orange. First flagellomere 2 times as long as pedicel. Arista with
short rays, longest dorsal rays usually about twice as long as basal width of
arista; ventral rays shorter. Frons completey silvery, less dense in front of
ocellar triangle, with short scattered darker setulae. Face orange. Genal
setulae and genal seta black, well developed.

Thorax.
Postpronotal lobe pale greyish white; without spot. Scutum, ground colour
greyish, with darker stripes and streaks but no distinct black spots except
near intra-alar and prescutellar acrostichal setae; prescutellar markings
white, separated by pale area or merged; with silver-grey microtrichosity and
pilosity. Scapular setae black. Scutellum pale yellow, basally with two
distinct black spots, apically with three separate black spots, extending to
basal third. Subscutellum completely black. Anepisternum lower half
yellow-brown, upper half white; two black anepisternal setae, setulae white,
upper margin with black setulae.

Legs.
Pale yellowish brown; setation mixed black and white. Fore femur with ventral
setae black.

Wing.
Bands mainly brown. Anterior apical and discal bands not separate; subapical
band separate; posterior apical band absent; crossvein R-M at, or just distal
to, middle of discal medial cell.�

Abdomen.
greyish, lateral margin and tergite 5 largely with orange tinge; pilosity mixed
black and white. Tergites with darker spots.

Female

As
male except for the following characters. Frons yellow-brown, weakly silvery.
Face white, lower margin yellow. Oviscape about as long as preabdomen. Aculeus�
at least ten times longer than wide, tip bilobed and lateral margin slightly
sinuate.

(Description
after De Meyer & Copeland, 2005)
